# Impact of PKCε downregulation on autophagy in glioblastoma cells

**DOI:** 10.1186/s12885-018-4095-1

**Published:** 2018-02-13

**Authors:** Ewa Toton, Aleksandra Romaniuk, Natalia Konieczna, Johann Hofmann, Jan Barciszewski, Maria Rybczynska

**Affiliations:** 1 Department of Clinical Chemistry and Molecular Diagnostics, Poznan University of Medical Sciences, Przybyszewskiego 49 St., 60-355 Poznan, Poland; 2 Biocenter, Division of Medical Biochemistry, Innsbruck Medical University, Innrain 80-82, A-6020 Innsbruck, Austria; 3 NanoBioMedical Center, Adam Mickiewicz University in Poznan, Poznan, Poland; 4 Institute of Bioorganic Chemistry, Polish Academy of Sciences, Poznan, Poland

**Keywords:** Autophagy, Protein kinase C epsilon, Gliomas

## Abstract

**Background:**

Several efforts have been focused on identification of pathways involved in malignancy, progression, and response to treatment in Glioblastoma (GB). Overexpression of PKCε was detected in histological samples from GB, anaplastic astrocytoma, and gliosarcoma and is considered an important marker of negative disease outcome. In multiple studies on GB, autophagy has been shown as a survival mechanism during cellular stress, contributing to resistance against anti-cancer agents. The main object of this research was to determine the influence of PKCε downregulation on the expression of genes involved in autophagy pathways in glioblastoma cell lines U-138 MG and U-118 MG with high PKCε level.

**Methods:**

We conducted siRNA-mediated knockdown of PKCε in glioblastoma cell lines and studied the effects of autophagy pathway. The expression of autophagy-related genes was analyzed using qPCR and Western blot analysis was carried out to assess protein levels. Immunostaining was used to detect functional autophagic maturation process.

**Results:**

We found that these cell lines exhibited a high basal expression of autophagy-related genes. Our results suggest that the loss of PKCε contributes to the downregulation of genes involved in autophagy pathways. Moreover, most of the changes we observed in Western blot analysis and endogenous immunofluorescence experiments confirmed dysfunction of autophagy programs. We found that knockdown of PKCε induced a decrease in the expression of Beclin1, Atg5, PI3K, whereas the expression of other autophagy-related proteins mTOR and Bcl2 was increased. Treatment of control siRNA glioma cells with rapamycin-induced autophagosome formation and increase in LC3-II level and caused a decrease in the expression of p62. Additionally, PKCε siRNA caused a diminution in the Akt phosphorylation at Ser473 and in the protein level in both cell lines. Moreover, we observed reduction in the adhesion of glioblastoma cells, accompanied by the decrease in total FAK protein level and phosphorylation.

**Conclusions:**

Effects of down-regulation of PKCε in glioma cells raised the possibility that the expression of PKCε is essential for the autophagic signal transduction pathways in these cells.

Thus, our results identify an important role of PKCε in autophagy and may, more importantly, identifyit as a novel therapeutic target.

## Background

Glioblastoma (GB), WHO grade IV, is considered to be the most rapidly progressive and invasive type of primary central nervous system tumor. GB occurs more frequently than other malignant brain tumors and has a poor prognosis with a median survival of less than 1 year [1]. Even with aggressive treatment using surgery, radiation, and chemotherapy, recurrences occur in almost all cases. The main features of GBs are genomic instability, abnormal cellular proliferation, high infiltration, intensive angiogenesis, and resistance to chemotherapy [2]. Previous studies have indicated that GBs are characterized by a heterogeneous population of cells. This heterogeneity probably arose from a series of mutations. The highly unstable nature of the cells is commonly caused by mutation of tumor suppressor genes, of following proteins: p53, p21, p16, and PTEN. Several intracellular pathways have been associated with growth and survival of GB [3, 4].

Protein kinase C (PKC) is a multigene family of phospholipid-dependent serine-threonine kinases that are involved in numerous signal transduction cascades. Overexpression of one of the 11 known PKC isozymes, PKCε, is a hallmark of human glioma [5] and acts in proliferation, differentiation, adhesion, migration, gene expression, and apoptosis [6]. PKCɛ was found to be oncogenic in rat fibroblasts and rat colonic epithelial cells in in vitro and in vivo conditions. Rat colonic epithelial cells with overexpressed PKCε exhibited that Raf-1/mitogen-activated protein kinase (MAPK) to be responsible for the PKCε-induced transformation [7]. There is increasing evidence to indicate that upregulation of PKCε in GB is associated with tumor aggressiveness and is widely implicated in malignant transformation [8]. However, its role in autophagy remains unclear. Autophagy is regarded both as cell death and cell survival mechanism, which depends on the environmental conditions and cellular status.

Autophagy is a highly conserved degradation pathway whose primary task is to recycle cellular components and degrade long-lived proteins. The process of autophagy involves the formation of double-membrane autophagosomes [9]. Numerous studies have shown that in gliomas, autophagy functions mainly as a survival mechanism, stimulated through cellular damage, caused by chemotherapeutic treatment and through various metabolic stresses, such as nutrient or growth factor deprivation [10, 11]. Li et al. investigated the effect of autophagy inhibition at different stages on cytotoxicity of autophagy inducers in glioblastoma cells. Obtained data indicate that inhibition of late steps of autophagy sensitized GB cells to arsenic trioxide (ATO) treatment and induced stronger apoptosis [12]. Okhrimenko et al. found that the knockdown of PKCε selectively reduced the expression of Akt and induced cell apoptosis in the glioma cell lines and primary cultures. Moreover, overexpression of PKCε protects glioma cells from the apoptosis induced by TNF-related apoptosis-inducing ligand (TRAIL) [13]. Akt (PKB) is a family of serine-threonine kinases which was first identified in 1991 independently by three different groups [14]. Akt mediates carcinogenesis and tumor progression mainly through promoting cell survival and inhibiting apoptosis in a variety of cellular systems including gliomas [15]. The survival effects of Akt are exerted by inhibiting apoptosis through phosphorylation and inactivation of several targets, including Bad, forkhead transcription factors, c-Raf, and caspase-9 [16]. Akt also plays a critical role in cell growth by directly phosphorylating mTOR in a rapamycin-sensitive complex containing raptor [17, 18]. The activity of Akt is regulated by phosphorylation on Thr308 by PDK-1 and on Ser473 by PDK-2 [19].

Recently, Okhrimenko et al. have shown that silencing of PKCε-induced apoptosis in glioma stem cells (GSCs), suggesting that in these cells also PKCε contributes to survival and their ability to self-renew [15]. Also, the functional relationship between autophagy and apoptosis is complex, and that one can stimulate or inhibit the other [20]. These data raised the possibility that the PKCε is essential for the survival of glioblastoma cells. We were trying to determine whether silencing the expression of *PRKCE* via RNA interference (siRNA) can affect the lifespan of cancer cells with overexpressed PKCε by autophagy pathway interruption. The study also contains the assessment of the effect of different compounds (rapamycin, 3-methyladenin) on autophagy in cells with downregulated oncoprotein PKCε.

## Methods

### Cell line and culture conditions

The four human glioblastoma tumor cell lines such as T98-G, LN-18, U-118 MG, and U-138 MG were obtained from the American Type Culture Collection (Rockville, MD) and were grown in Eagle’s Minimum Essential medium supplemented with 10% fetal bovine serum (U-138 MG, LN-18) or Dulbecco’s Modified Eagle medium with 10% fetal bovine serum (U-118 MG, T98-G). All glioma cell lines were kindly provided by Prof. Jan Barciszewski (NanoBioMedical Center, Adam Mickiewicz University in Poznan and Institute of Bioorganic Chemistry, Polish Academy of Sciences, Poznan, Poland). The cells’ age did not exceed 15 passages.

The HeLaPKCεA/E subline was derived from parental HeLa wild-type cells, using transfection as previously described [21]. Created cell line shows constitutively active PKCε without activators such as TPA (12-O-Tetradecanoylphorbol-13-acetate). The HeLaPKCεA/E cells were grown in DMEM medium supplemented with 10% Tet-Approved fetal bovine serum (Clontech, Mountain View, USA), 100 μg/ml geneticin, and 100 μg/ml hygromycin (both from Roche Diagnostics, Mannheim, Germany). All cells were propagated in a humidified atmosphere containing 5% CO_2_ at 37 °C.

### siRNA transfection

Two glioblastoma cell lines (U-138 MG and U-118 MG) were transfected with small interfering RNA against PKCε (PKCε siRNA) and non-targeting siRNA (Control-siRNA) (Santa Cruz Biotechnology, CA, USA). Transfections were performed at approximately 60% confluence in six-well plates using TransIT-siQUEST® Transfection Reagent (Mirus Bio, Madison, WI, USA) according to the manufacturer’s protocol. Briefly, 1 × 10^5^ cells per well were seeded in complete growth medium the day before transfection. The siRNA-transfection reagent complex was prepared in a medium without FBS and incubated with cells for 72 h at 37 °C, 5% CO_2_. The final concentration of the siRNA was 25 nM. In each experiment, untreated controls and mock-transfected cells were included. Cells were collected 72 h after transfection.

### Detection of *PRKCE* mRNA level by q-PCR

The effect of siRNA (25 nM, treatment for 72 h) on the expression of the *PRKCE* gene in two cell lines such as U-118 MG and U-138 MG was assessed using q-PCR. Briefly, total RNA was extracted with TRI Reagent (Sigma-Aldrich, St Louis, MO, USA) according to the method of Chomczynski and Sacchi [22]. RNA concentration was quantified by measuring optical density (OD) at 260 nM using a BioPhotometer Plus (Eppendorf, Hamburg, Germany). The Transcriptor First Strand cDNA Synthesis Kit (Roche Diagnostics, IN, USA) was used to cDNA synthesis, using 0.5 μg of total RNA and oligo(dT) primer. The real-time polymerase chain reaction for PKCε expression analysis was carried out using LightCycler 96 with specific primers for *PRKCE* gene: F—5′-TCAGCAAGGAGGCTGTCA-3′, R—5′-ATGGGTGCTGCTTGATGG-3′ designed with Universal Probe Library software (Roche Diagnostics, IN, USA). Amplification products of individual gene transcripts were detected via intercalation of the fluorescent dye SYBR Green (LightCycler FastStart DNA Master SYBR Green 1 kit, Roche Diagnostics, IN, USA). Cycling conditions for all amplicons were as follows: initially 95 °C for 10 min, followed by 45 cycles at 94 °C for 20 s, 57 °C for 20 s, and 72 °C for 20 s. All cycling reactions were performed in the presence of 2.5 mM MgCl_2_. Gene-specific products were confirmed by melting curve analysis. The expression was normalized by the expression of the housekeeping gene *GAPDH*: F—5′-TTCGTCATGGGTGTGAACC-3′, R—5′-GATGATGTTCTGGAGAGCCC-3′.

### qPCR array of autophagy gene expression

The effect of silencing *PRKCE* gene and rapamycin (300 nM) and 3-MA (5 mM) treatment for 24 h on the expression of genes involved in the autophagy pathways was assessed using Real Time Primers ready Human Autophagy Primer Library 96 (HATPL-1) (Biomol GmbH, Hamburg, Germany). The cDNA was prepared as described above. q-PCR was performed using the FastStart Essential DNA Green Master (Roche Diagnostics, IN, USA). The thermal cycle profile was 95 °C for 10-min initial denaturation (hot start) followed by 94 °C for 20 s, 58 °C for 20 s, and 72 °C for 20 s for 50 cycles. Beta-2-microglobulin (*B2M)* expression was used as an internal control as previously described [21]. Relative quantities (RQ) of gene expression for sample comparison was calculated using the comparative threshold cycle (CT) method (also known as the 2 ΔΔCT method). Experiments were performed in duplicates, and results show mean values normalized to the expression of indicated genes in control cells (Control siRNA-treated cells or Control cells). Results were analyzed and shown using GENE-E (Broad Institute, Cambridge, UK).

### Immunodetection

**Fig. 1:**
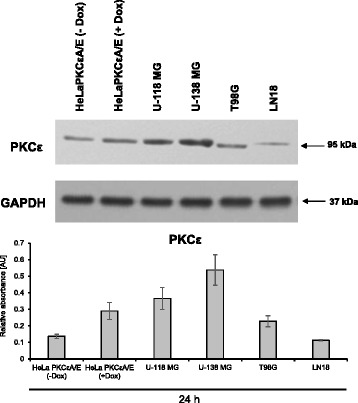
PKCε protein levels in HeLaPKCεA/E and glioblastoma cell lines. Protein extracts from HeLaPKCεA/E (−Dox), HeLaPKCεA/E (+Dox), U-118 MG, U-138 MG, T98G, LN18 cell lines were subjected to Western blot analysis using antibodies against PKCε and GAPDH. HeLaPKCεA/E cells with doxycycline-induced expression of PKCε (+Dox) and without doxycycline treatment (−Dox) served as a reference cell lines with low and high expression of PKCε, respectively. The average relative absorbance of three independent experiments is presented in a bar graph

U-118 MG and U-138 MG cells were treated with PKCε siRNA (25 nM) or control siRNA (25 nM) (both from Santa Cruz Biotechnology, CA, USA) for 72 h and rapamycin (300 nM) or 3-methyladenine (5 mM) (both from Sigma, St Louis, MO, USA) for 24 h. Whole cell extracts were prepared using a modified RIPA lysis buffer [21]. The protein concentration was measured using a Bradford assay (Sigma, Munich, Germany) and 30 μg–60 μg of protein extracts were separated by electrophoresis on 4–20% Mini-PROTEAN Stain-free gels (BioRad, Hercules, CA). Standard immunodetection was performed, using a PVDF membrane (Pierce Biotechnology, Rockford, USA). The following antibodies were used for detection: anti-PKCε, anti-BECN1, anti-Bcl-2, anti-FAK, anti-pFAK (Tyr-397), anti-pFAK (Tyr 576/577), anti-GAPDH, anti-rabbit IgG-HRP (all from Santa Cruz Biotechnology, CA, USA), anti-MAPLC3β (Sigma, St Louis, MO, USA), anti-ATG5, anti-mTOR, anti-SQSTM1/p62, anti-Akt and anti-pAkt (Ser473) (from Cell Signaling Technology, Danvers, MA, USA). The 1:1000 dilution of primary antibody was used. The proteins were visualized using a SuperSignal® West Pico Chemiluminescent Substrate and CL-X Posure™ film (Pierce Biotechnology, Rockford, USA). Densitometric scanning of the blots was quantified with LabWorks software version 4.6 using a BioImaging Systems EpiChemi^3^ Darkroom (UVP, Inc., Upland, CA, USA). The scan of one out of three performed experiments is shown (Figs. 1, 3, 6, 7, 8, 9, 11 and 12).

### Autophagosome visualization

The study was carried out in 6-well culture dishes with glass coverslips. HeLaPKCεA/E, cells (5 × 10^4^/ml) were incubated overnight to reach sedimentation and then were treated with 2 μg/ml Dox for 24 h and 300 nM rapamycin for 24 h. U-138 MG and U-118 MG cells (5 × 10^4^/ml) were incubated overnight to reach sedimentation and then were treated with PKCε siRNA (25 nM) or control siRNA (25 nM) for 72 h and rapamycin (300 nM) for 24 h. For autophagosome detection, cells were stained with primary antibody rabbit anti-MAPLC3β (Sigma, St Louis, MO, USA, 1:200) as previously described [21]. The cultures were incubated with Alexa Fluor 488 goat anti-rabbit IgG secondary antibody fluorescent dye (Life Technologies, Molecular Probes, Grand Island, NY, USA) and recorded at 488 nm using a fluorescent microscope (Carl Zeiss, Gőttingen, Germany).

### Attachment assay

After transfection, cells were washed with PBS, harvested by trypsin, resuspended in fresh medium and counted. For attachment assay, cells were seeded in 6-well culture plates (7,5 × 10^4^/ml) coated with Matrigel (Corning Life Sciences, NY, USA). Culture plates were prepared according to the instructions of the manufacturer. Next, the cells were incubated at 37 °C. During microscopic observation, photographs were taken (Axiovert 40 CFL, Zeiss, Göttingen, Germany) after 60 and 180 min of incubation. A representative picture of two performed experiments is shown (Fig. 12a and b). The percentage of adhesion cells was calculated as the ratio of adhesion cells to total cells counted. We counted a minimum 100 cells per field and at least three areas in each well.

### Statistical analysis

All results are means from three or five separate experiments unless specified otherwise. Statistical analysis was performed by one-way ANOVA (GraphPad Prism, San Diego, CA). *P* < 0.05 was considered as significant difference.

## Results

### Level of PKCε in glioblastoma cell lines

Several efforts have been focused on identification of pathways involved in malignancy, progression, and response to treatment in GB [23, 24]. It has been suggested that high PKCε level contributes to cancer development and increases the ability of tumor cells to metastasis [25–27]. Overexpression of PKCε was detected earlier in cancer cells from astrocytoma, GB, and gliosarcoma [5, 28]. In line with others [15, 29] we also find PKCɛ is highly expressed in glioblastomas. In this study, we assessed the level of PKCε protein in selected glioblastoma cell lines as follows: U-118 MG, U-138 MG, T98G, and LN18 after 24 h of incubation. The HeLaPKCεA/E subline (with overexpressed PKCεA/E) containing a constitutively active rat PKCε induced by 24 h treatment with 2 μg/ml doxycycline (Dox) served as a positive control in Western blot analysis [27]. The level of PKCε protein is varied depending on the cell line. The U-138 MG cells presented the highest level of PKCε protein among studied cell lines. Two GB cell lines such as U-138 MG and U-118 MG compared to doxycycline-induced HeLaPKCεA/E cells (with overexpressed PKCεA/E) exhibited higher PKCε protein level, whereas LN18 and T98G cell lines presented lower expression level (Fig. 1). To verify the significance of high PKCε level in gliomas, we chose two cell lines for further analysis as follows: U-138 MG and U-118 MG.

### Assessment of autophagy gene expression in U-138 MG and U-118 MG

PKCε is potentially related to pathways that promote survival and inhibit apoptosis of cancer cells [30]. The main process involved in cell death and survival is autophagy. It plays a dual role in tumor development. On the one hand, it enables tumor cells to survive under adverse conditions. On the other hand, it limits the cell growth and genomic instability. These characteristics indicate that autophagy may represent a novel therapeutic target [31]. It has been reported that some members of PKC family (PKCα, PKCδ and PKCθ) are involved in autophagy induction [32–35]. Furthermore, several studies have used pharmacological activators or inhibitors of PKCs to establish their role in autophagy. Safingol induces autophagy in solid tumor cells through inhibition of PKC (βI, δ, and ε) and PI3-kinase pathway [36].

**Fig. 2:**
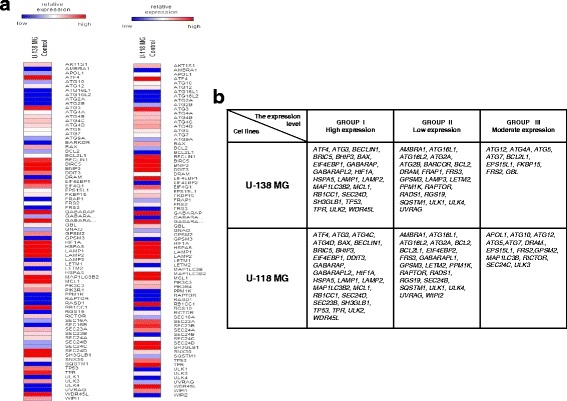
Basal expression of autophagy-related genes in U-138 MG and U-118 MG cell lines. **a** Heat maps of differentially expressed autophagy-related genes in U-138 MG and U-118 MG cell lines with high basal PKCε expression levels. Each row represents a relative gene expression normalized to Beta-2-microglobulin gene (*B2M*) expression. Red color indicates genes with high relative expression, and blue color indicates genes with low relative expression. Results show mean values from three independent experiments performed in duplicates. **b** Classification of autophagy genes according to the relative expression level in U-138 MG and U-118 MG cell lines. Group I contains genes with the highest expression, Group II with the lowest, and Group III with moderate expression levels

However, since there are no reports about the connection between PKCε expression and autophagy process, we assessed the basal expression of autophagy-related genes in glioblastoma cell lines with high PKCε level (Fig. 2a). Analysis of the gene expression profile indicates high expression of autophagy-related genes and similar transcriptional profiles in U-138 MG and U-118 MG cell lines. Depending on the initial level of relative expression, we classified autophagy genes into three groups: I—genes with high expression, II—genes with low expression, III—genes with moderate expression level (Fig. 2b). We observed consistent alterations in the expression of certain genes involved in the activation of autophagy (upregulation of, e.g., *BECLIN1, MAP1LC3B2*) as well as genes related to autophagy inhibition (downregulation of, e.g., *BCL2, RAPTOR*). Our present data suggest that high level of PKCε expression may be correlated with upregulation of autophagy process (Figs. 1 and 2).

### Downregulation of *PRKCE* expression

**Fig. 3:**
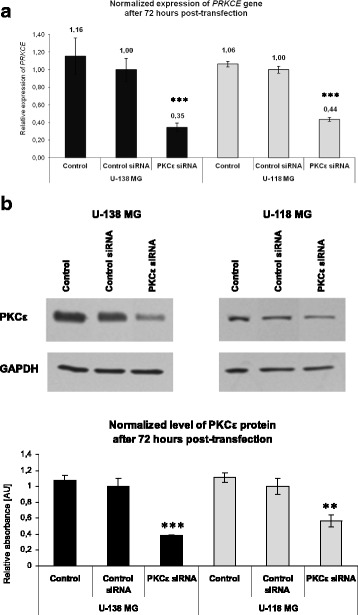
The effect of *PRKCE* silencing on U-138 MG and U-118 MG cells. Cells were harvested 72 h after transfection with non-targeting siRNA (Control siRNA) or PKCε siRNA. The downregulation of *PRKCE* mRNA expression (**a**) and PKCε protein level (**b**) are shown. The bar graphs present average values of three independent experiments. ** *P* < 0.01, *** *P* < 0.001

It was reported that glioblastoma cells have distinct transcriptional profiles depending on molecular subtype and grade [37, 38]. The expression profile of studied glioblastoma cell lines with high PKCε level suggests basal upregulation of autophagy pathways. To determine the role of PKCɛ in the regulation of autophagy pathways in glioma cells, we used the siRNA against PKCε (PKCε siRNA). We obtained about 65% and 56% decrease in mRNA (Fig. 3a) and 62% and 42% reduction in protein content for U-138 MG and U-118 MG cells, respectively (Fig. 3b).

### Assessment of autophagy gene expression in U-138 MG and U-118 MG after PKCε downregulation

Next, we examined whether siRNA silencing of PKCε can change the expression of genes involved in autophagy pathways, using qPCR arrays. Considering the baseline cellular response and the risk of off-target effects, we initially compared gene expression in non-treated control cells; and non-targeting siRNA-treated cells. At the molecular level, autophagy is controlled by AuTophaGy-related genes (ATG) and their respective Atg proteins [39]. The analysis exhibited that expression level of a number of genes was reduced after the treatment with non-targeting siRNA (U-138 MG cells: *ULK3, LAMP1, EIF4G1, SEC24C*; U-118 MG cells: *ATG16L2, AMBRA1, UKLK1, LAMP1, WDR45L, FRS3, ATG2A, RAPTOR, SEC23A, SEC24C, AKT1S1, ATG2B, MCL1, SEC24A*) as well as some genes were upregulated (U-138 MG cells: *UVRAG, BCL2L1, ATG16L1, MAP1LC3B3, WIPI2, BARKOR, ATG12, DRAM, SQSTM1*; U-118 MG cells: *BAX, DRAM, GPSM2, EIF4EBP2, ATG12, BCL2L1, SEC24B, MAP1LC3B, SQSTM1, ATG16L1, LETM2*). Due to the risk of nonspecific effect, these genes were excluded from further gene expression analysis (data not shown).

**Fig. 4:**
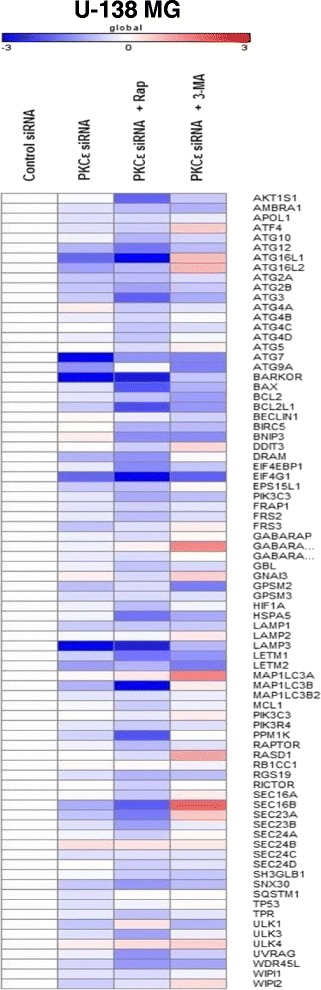
Changes in autophagy gene expression in U-138 MG cells after PKCε downregulation and rapamycin or 3-MA treatment. The heat map shows the relative expression of human autophagy genes in U-138 MG cells transfected for 72 h with PKCε siRNA (PKCε siRNA) and in cells transfected and then treated for 24 h with rapamycin (PKCε siRNA + Rap) or 3-MA (PKCε siRNA + 3-MA). Each row represents an average gene expression normalized to the expression of the indicated gene in cells transfected with non-targeting siRNA (Control siRNA). Red color indicates genes that were upregulated, and blue color indicates genes that were downregulated. White indicates genes whose expression is unchanged in analyzed cells as compared to Control siRNA. Experiments were performed three times in duplicates

Activation of autophagy involves a series of steps starting with the initiation of the phagophore. It is mediated by the Atg1/ULK kinase complex and is inhibited by mTORC1. During nucleation, the Atg proteins are recruited to the phagophore via complex integrated by Beclin1, class III phosphatidylinositol 3-kinase (PI3K), and several other proteins [40]. As shown in Fig. 4, we observed global downregulation of autophagy-related genes after PKCε silencing in U-138 MG cells, compared with control siRNA-treated cells. The analysis of the panel of autophagy-related genes demonstrated that knockdown of PKCε significantly decreased expression of *ATG7, ATG12, ATG16L1, ATG9A, BARKOR, DRAM, EIF4G1 LAMP3, LETM2, ULK1* and *MAP1LC3B* genes; however, there was also a group of genes that were slightly induced, e.g.*, ATG4A, BNIP3, GNAI3, SEC24B,* and *ULK4*. Data revealed that the silencing of PKCε in U-138 MG decreased the expression of genes involved in the formation of phagophore and autophagosome.

To define the pathway by which knockdown of PKCɛ influences autophagy in glioma cells, we treated transfected cells with autophagy modulators: rapamycin and 3-methyladenine (3-MA). Rapamycin is a macrocyclic antibiotic, which is originally identified as a fungicide and immunosuppressant. Studies revealed that rapamycin specifically inhibits its target, mammalian target of rapamycin (mTOR), thereby enhancing autophagy [41]. 3-Methyladenine suppresses autophagy via the inhibition of type III phosphatidylinositol 3-kinases (PI3K). PI3K is essential in initiating autophagy through the recruitment of Atg proteins at the phagophore [42]. Interestingly, when rapamycin was used in U-138 MG PKCε siRNA cells, the expression of autophagy-related genes was significantly decreased relative to both control siRNA and PKCε siRNA cells. Downregulation was observed for the following genes: *AKT1S1, ATG10, ATG12, ATG16L1, ATG3, ATG4A, ATG4B, ATG4C, ATG4D,BARKOR, BAX, BCL2L1, BNIP3, EIF4G1, PIK3C3, HSPA5, LAMP3, LETM1, MAP1LC3B, PPM1K, SEC16B, SEC23A, SEC23B, SNX30, ULK3, UVRAG,* and *WDR45L*. In contrast, the treatment with 3-methyladenine (3-MA) upregulated the expression of *ATF4, ATG16L1, ATG16L2, DDIT3, GABARAPL1, GNAI3, MAP1LC3A, RASD1, SEC16B, SEC23A, ULK4,* and *WIPI2* genes in U-138 MG PKCε siRNA cells compared to control siRNA and PKCε siRNA cells (Fig. 4).

**Fig. 5:**
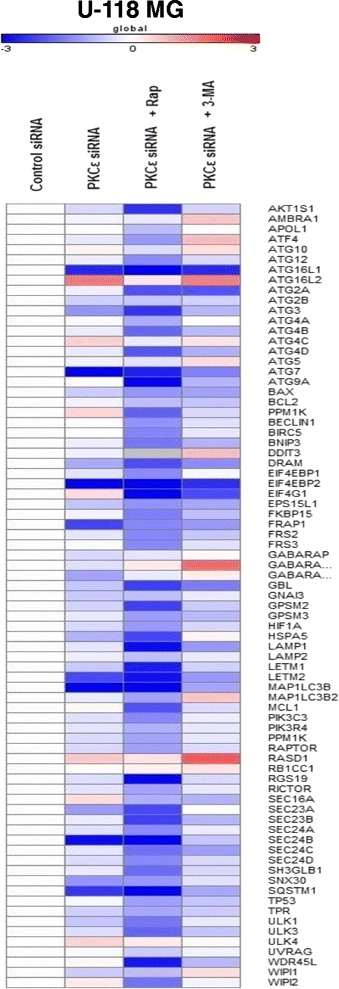
Changes in autophagy gene expression in U-118 MG cells after PKCε downregulation and rapamycin or 3-MA treatment. The heat map shows the relative expression of human autophagy genes in U-118 MG cells transfected for 72 h with PKCε siRNA (PKCε siRNA) and in cells transfected and then treated for 24 h with rapamycin (PKCε siRNA + Rap) or 3-MA (PKCε siRNA + 3-MA). Each row represents an average gene expression normalized to the expression of the indicated gene in cells transfected with non-targeting siRNA (Control siRNA). Red color indicates genes that were upregulated, and blue color indicates genes that were downregulated. White indicates genes whose expression is unchanged in analyzed cells as compared to Control siRNA. Experiments were performed three times in duplicates

Next, we analyzed the expression of autophagy-related genes in U-118 MG cells in the same experimental model: the PKCε siRNA transfection followed by rapamycin or 3-MA treatment. The analysis demonstrated that siRNA silencing of *PRKCE* in U-118 MG cells caused a significant decrease in numerous autophagy-related gene expression compared to control siRNA-treated cells (Fig. 5). Genes *ATG16L1, ATG 3, ATG7, DRAM, EIF4EBP2, FRAP1, LETM2, MAP1LC3B, SEC23A, SEC24B, SNX30,* and *SQSTM1* were significantly downregulated. Silencing of *PRKCE* gene in U-118 MG cells also resulted in upregulation of *ATG16L2, ATG4C, EIF4G1, PPM1K, RASD1, SEC16A* and *ULK4* genes.

Similar to the results for U-138 MG cell line, rapamycin treatment of U-118 MG cells after PKCε downregulation resulted in the inhibition of expression of most of the analyzed genes (*AKT1S1, ATF4, ATG12, ATG16L1, ATG2A, ATG3, ATG4A, ATG4B, ATG4D, ATG7, ATG9A, BAX, PPM1K, BECLIN1, BIRC5, BNIP3, DRAM,EIF4GP1 EIF4GP2, EIF4G1, EPS15L1, FKBP15, FRAP1, FRS2, FRS3, GBL, GPSM2, HSPA5, LAMP1, LETM1, LETM2, MAP1LC3B, MCL1, PIK3C3, RGS19, SEC23A, SEC23B, SEC24B, SEC24C, SH3GLB1, SQSTM1, ULK1, ULK3, WDR45L, WIPI2*). Moreover, 3-MA treatment induced expression of several genes (*AMBRA1, ATF4, ATG10, ATG16L2, ATG5, DDIT3, GABARAPL1, MAP1LC3B2, RASD1*) (Fig. 5).

After PKCε siRNA treatment we observed downregulation of genes involved in autophagy pathways. In addition, we showed that positive autophagy regulator, rapamycin caused an increase in downregulation of autophagy-related genes in PKCε siRNA cells (Figs. 2, 4, and 5).

### Alterations of autophagy-related proteins after PKCε downregulation

In order to examine the role of PKCε in autophagy pathways in glioma cells, we evaluated the impact of siRNA silencing of PKCε on the level of crucial autophagic proteins. Autophagy is induced by an initial membrane nucleation step that requires the ULK1 complex and a class III phosphoinositide 3-kinase complex including Beclin1. Beclin1 is a protein that promotes the formation of the Beclin-1-Vps34-Vps15 complex triggering the autophagy protein cascade [43]. This protein complex recruits many of the key autophagic proteins required for proper initiation of autophagosome formation, e.g. ubiquitin-like conjugation systems Atg5-Atg-12 and LC3 [44]. Several studies have demonstrated that mTOR (mammalian target of rapamycin) kinase is a central checkpoint that downregulates autophagy [45–47]. In our study, we classified the crucial protein markers of autophagy into three groups: I—proteins involved directly in autophagy initiation (mTOR, PI3K, Beclin1), II—proteins engaged in the formation of a double-membrane structure known as autophagosome (Atg5, LC3-II), and well-characterized autophagosome substrate (p62/SQSTM1), III—protein that regulates autophagy process (Bcl-2).

**Fig. 6:**
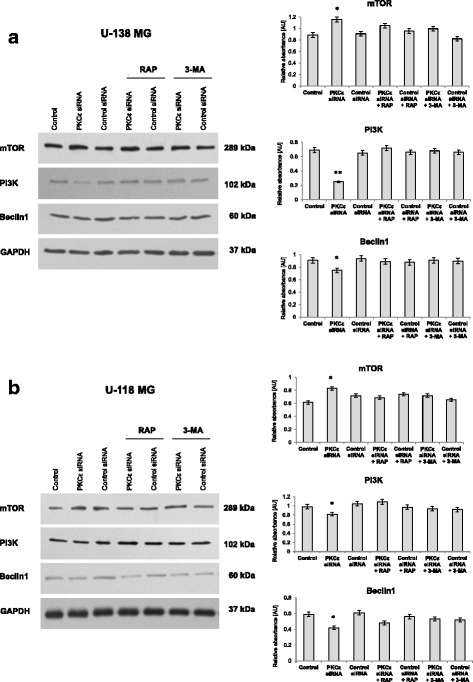
Influence of PKCε downregulation and rapamycin or 3-MA treatment on protein level directly involved in autophagy activation (mTOR, PI3K, Beclin1). **a** U-138 MG and **b** U-118 MG cells were transfected for 72 h with PKCε siRNA (PKCε siRNA) and non-targeting siRNA (Control siRNA) and then were treated for 24 h with rapamycin (300 nM) (PKCε siRNA + Rap) or 3-MA (5 mM) (PKCε siRNA + 3-MA). GAPDH was used as a loading control and as an internal standard. Representative blots are shown. In case of mTOR the order of bands has been changing, due to a different final construct of probe layout. The densitometric analysis represents means ±SD of three independent experiments. * *P* < 0.05, ** *P* < 0.01 statistically significant compared to Control siRNA

As presented in Fig. 6a and b knockdown of PKCε drastically reduced the level of PI3K and Beclin1 protein in U-138 MG and U-118 MG cells compared to control siRNA cells. Moreover, in those cells, the level of anti-autophagic mTOR was increased.

Next, we analyzed the level of these same proteins in U-138 MG and U-118 MG cells after siRNA silencing of *PRKCE* followed by 24-h treatment with rapamycin (300 nM) or 3-MA (5 mM). In both glioblastoma cell lines, the analysis demonstrated no significant changes in the level of PI3K, Beclin1, and mTOR compared to control siRNA cells (Fig. 6a and b).

**Fig. 7:**
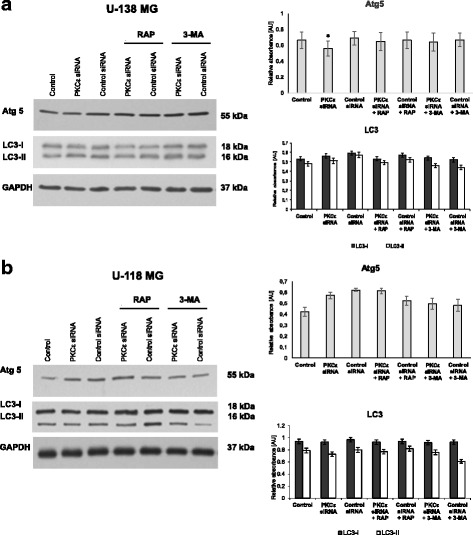
Effect of PKCε downregulation and rapamycin or 3-MA treatment on proteins engaged in the formation of a double-membrane structure known as autophagosome (Atg5, LC3-II). **a** U-138 MG and **b** U-118 MG cells were transfected for 72 h with PKCε siRNA (PKCε siRNA) and non-targeting siRNA (Control siRNA) and then were treated for 24 h with rapamycin (300 nM) (PKCε siRNA + Rap) or 3-MA (5 mM) (PKCε siRNA + 3-MA). GAPDH was used as a loading control and as an internal standard. Representative blots are shown. The densitometric analysis represents means ±SD of three independent experiments. * *P* < 0.05, statistically significant compared to Control siRNA

Thereafter, we assessed changes in the second group of autophagy markers consisted of proteins engaged in the formation of a double-membrane structure known as autophagosome (Atg5, LC3-II). Atg5 protein creates a complex with Atg12 and mediates LC3 conversion and by that autophagosome formation [40]. As shown in Fig. 7a and b PKCε downregulation in U-138 MG and U-118 MG cells led to decreased levels of Atg5.

Next, we analyzed the accumulation of LC3-II, the lipidated form of LC3 associated with the autophagosome membrane. The amount of LC3-II reflects the number of autophagosomes [45]. As shown in Fig. 7a and b, we observed no significant changes in the level of LC3-II in PKCε siRNA cells compared to control siRNA cells. Moreover, our study showed alternations in the level of LC3-II protein under the influence of modulators of autophagy process (rapamycin and 3-MA) in control siRNA cells (Fig. 7b).

p62, also known as SQSTM1/sequestome 1, serves as a link between LC3 and ubiquitinated substrates and is efficiently degraded by autophagy. Autophagic suppression correlates with an increased p62 level, and similarly, autophagic activation correlates with a decreased p62 level. Thus, the measurement of the cellular p62 level appears to correlate well with other markers of autophagic flux. Therefore, is recommended that to monitor autophagy use combination of p62 level and LC3-II turnover [48].

**Fig. 8:**
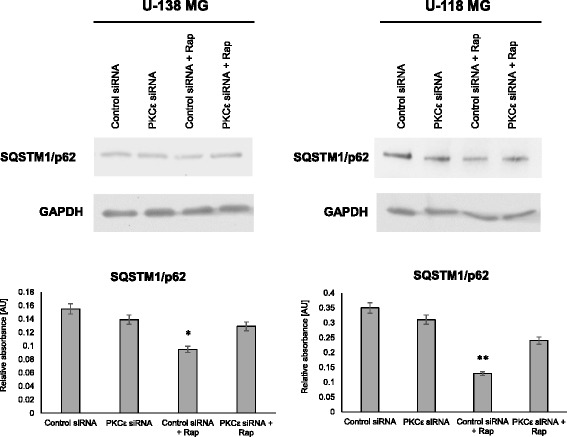
Modulation SQSTM1/p62 after *PRKCE* silencing and rapamycin treatment. **a** U-138 MG and **b** U-118 MG cells were transfected for 72 h with PKCε siRNA (PKCε siRNA) and non-targeting siRNA (Control siRNA) and then were treated for 24 h with rapamycin (300 nM). GAPDH was used as a loading control and as an internal standard. Representative blots are shown. The densitometric analysis represents means ±SD of three independent experiments. * *P* < 0.05, statistically significant compared to Control siRNA

As shown in Fig. 8 we observed lack of autophagy activation after treatment with rapamycin in PKCε siRNA cells, but in control siRNA cells there was a decrease in a p62 level that indicates autophagy activation. Our results suggest that PKCε siRNA transfection inhibited rapamycin-induced autophagy in both glioblastoma cell lines with overexpressed PKCε.

**Fig. 9:**
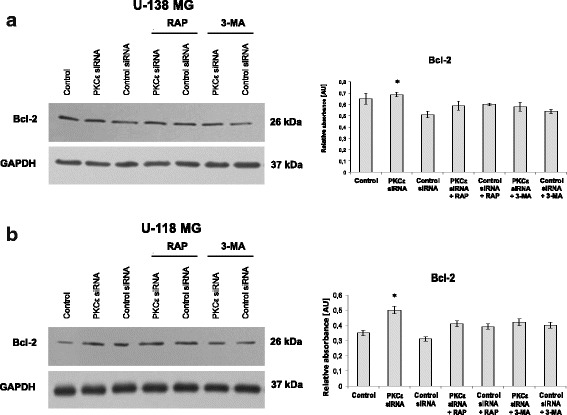
Effect of PKCε downregulation and rapamycin or 3-MA treatment on Bcl-2, a protein that regulates autophagy process. **a** U-138 MG and **b** U-118 MG cells were transfected for 72 h with PKCε siRNA (PKCε siRNA) and non-targeting siRNA (Control siRNA) and then were treated for 24 h with rapamycin (300 nM) (PKCε siRNA + Rap) or 3-MA (5 mM) (PKCε siRNA + 3-MA). GAPDH was used as a loading control and as an internal standard. Representative blots are shown. The densitometric analysis represents means ±SD of three independent experiments. * *P* < 0.05, statistically significant compared to Control siRNA

Next, we investigated the protein that takes part in the regulation of autophagy process—Bcl-2. As shown in Fig. 9a and b, knockdown of PKCε markedly enhanced the protein expression of Bcl-2 in both glioblastoma cell lines. Interaction Beclin1 with anti-apoptotic Bcl-2 prevents Beclin1 from assembling the pre-autophagosomal structure thus leads to inhibition of autophagy [49].

### Endogenous immunofluorescence of LC3

**Fig. 10:**
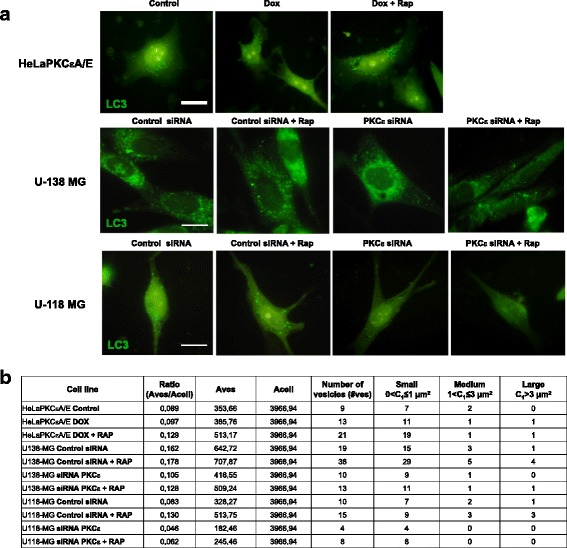
Endogenous immunofluorescence of LC3 after PKCε downregulation. **a** Cells were transfected with PKCε siRNA (PKCε siRNA) and non-targeting siRNA (Control siRNA) and then were treated with rapamycin (300 nM) for 24 h, as described in Autophagosome visualization section. Representative images from HeLaPKCεA/E, U-138 MG, and U-118 MG cells are shown. Scale bars equate to 10 μm. **b** AUTOCOUNTER analysis in imaging of U-138 MG and U-118 MG cells after siRNA silencing of PKCε (72 h) and Rapamycin (300 nM) treatment (24 h). The ratio between vesicle area and cell area (Aves/Acell), number of vesicles (#ves), and number of vesicles grouped, according to their area, into three defined classes (small: 0 < C1 ≤ 1 μm^2^; medium: 1 < C2 ≤ 3 μm^2^; large: C3 > 3 μm^2^) are reported. Cells were analyzed using a fluorescence microscope at 100× magnification and ImageJ JavaScript software, as described by Fassina et al. [50]

In order to understand autophagy dynamics, we assessed the endogenous immunostaining of LC3 protein. The HeLaPKCεA/E subline treated with doxycycline (Dox) and rapamycin (Rap) served as a positive control for autophagosome formation. As shown in Fig. 10a after treatment with rapamycin autophagosome accumulation has not been observed in glioblastoma cell lines treated with PKCε siRNA. In contrast treatment with rapamycin increased presence of autophagosome in control siRNA cells. The images recorded by fluorescence microscope were analyzed the variation of number and area of vesicles (represent autophagosomes) by AUTOCOUNTER (ImageJ JavaScript software) [50] (Fig. 10b). The received numerical data confirmed observed changes. The PKCε downregulation caused a decrease in number and size of vesicles and attenuation of the rapamycin effect. Observed changes had the same tendencies in all carried analysis.

### Evaluation of the level and phosphorylation of Akt

**Fig. 11:**
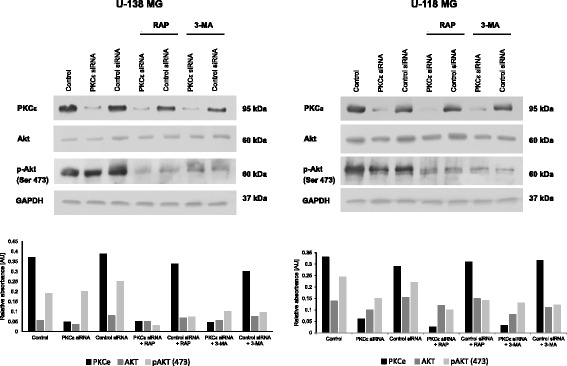
Knockdown of PKCɛ diminishes the level and phosphorylation of Akt in glioma cells. **a** U-138 MG and **b** U-118 MG were transfected for 72 h with PKCε siRNA and Control siRNA and then were treated for 24 h with rapamycin (300 nM) or 3-MA (5 mM). GAPDH was used as a loading control and as an internal standard. Representative blots are shown. The densitometric analysis represents means ±SD of three independent experiments

In the present study, after siRNA silencing of *PRKCE,* we analyzed the protein level and phosphorylation of Akt in U-138 MG and U-118 MG cells*.* As presented in Fig. 11 the silencing of PKCɛ significantly decreased the level of Akt compared to control siRNA cells in both tested cell lines. Next, we investigated phosphorylation of Akt at Ser 473. We observed that PKCɛ silencing decreased Akt phosphorylation compared to control siRNA cells. Additionally, rapamycin treatment resulted in the lower level of phosphorylation of Akt compared to control siRNA cells. The analysis of 3-MA treatment after knockdown PKCε demonstrated no significant changes in the Ser 473 phosphorylation of Akt compared to control siRNA cells. These results indicated that activation of the PKCε pathway could contribute to activations of PI3K/Akt pathway, through interactions between PKCε and Akt.

### Assesment of phosphorylation of focal adhesion kinase in U-138 MG and U-118 MG after PKCε downregulation

The cell adhesion to the extracellular matrix (ECM) plays a critical role in the regulation of essential cellular functions [51]. Focal adhesion kinase (FAK) mediates a number of signaling pathways associated with cell adhesion. The network contains many classes of signaling molecules. FAK is a cytoplasmic nonreceptor tyrosine kinase which transduces the matrix-dependent effects on cell proliferation, migration, and survival [52]. Phosphorylation of FAK at tyrosine 397 plays an essential role in tumor cell signaling and can be induced by growth factors and mechanical stress [53].

The link between PKCε and FAK was demonstrated by Heidkamp et al. [54]. They showed that overexpression of caPKCε (constitutively active PKCε) increased both Y397pFAK and total FAK.

**Fig. 12:**
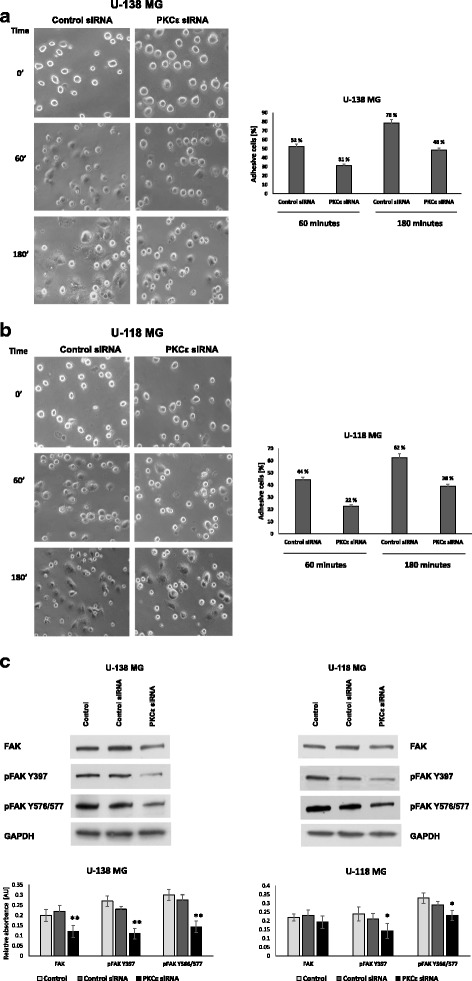
Effect of PKCε downregulation on the adhesion of glioblastoma cells. **a**. U-138 MG and **b** U-118 MG cells were transfected for 72 h with PKCε siRNA (PKCε siRNA) and Control siRNA and then were seeded in culture plates coated with Matrigel (Corning Life Sciences, NY, USA). The photos represent cell adhesion under the microscope at 100 x magnification field. **c** Evaluation of FAK expression in U-138 MG and U-118 MG cells with knockdown PKCε. Total protein expression and FAK phosphorylation at Tyr-397/Tyr-576/577 were assessed. GAPDH was used as a loading control and as an internal standard. Representative blots are shown. The densitometric analysis represents means ±SD of three independent experiments. * *P* < 0.05, statistically significant compared to Control siRNA

We performed a study on the role of PKCε in the adhesion of glioblastoma cells. The attachment assay was performed as shown in Fig. 12a and b, PKCε downregulation markedly diminished cell adhesion in both glioblastoma cell lines. In U-138 MG and U-118 MG cells, the PKCε siRNA transfection caused about 41% and 50% inhibition of cell adhesion after 60 min of incubation and 39% and 39% after 180 min of incubation, respectively (Fig. 12a and b). Western blot analysis revealed that PKCε downregulation significantly decreased phosphorylation of FAK at Tyr-397 and Tyr-576/577 in U-138 MG and U-118 MG cells. We also observed a reduction in total FAK protein level in both tested cell lines (Fig. 12c). Our study showed that downregulation of PKCε was lowering the adhesion of glioblastoma cells, accompanied by the decrease in total FAK protein level and FAK phosphorylation.

## Discussion

Numerous cancer processes are modulated by PKCε isoform activity, e.g., migration*,* adhesion, proliferation, and differentiation [31]. Studies reported that PKCε suppresses apoptosis and promotes tumor growth, and thus shows the greatest oncogenic potential of the PKC family [12]. High expression of PKCε was found in cancers of the breast, prostate, lung, leukemia, and glioblastomas [27, 31]. It has been shown that PKCε is overexpressed in many astroglial cell lines in comparison to normal astrocytes and contributes to establishing an aggressive phenotype [5, 55]. Since the role of PKCε in the apoptosis of glioma cells have been studied before [12], we aimed to target non-apoptotic death pathway-autophagy involved in survival, progression, and resistance of glioma cells [56]. To evaluate the correlation between high expression of PKCε and autophagy pathways in glioblastomas, we investigated the influence of *PRKCE* silencing on global expression of genes engaged in the autophagy process. In this study, we demonstrated that two glioblastoma cell lines such as U-138 MG and U-118 MG classified as grade IV astrocytoma have high basal level of PKCε protein.

We found that in selected glioblastoma cell lines, genes associated with autophagy induction are upregulated, suggesting that autophagy in those cells acts as a tumor-promoting factor. Increased basal levels of autophagy were also detected earlier in breast, pancreatic, and astrocytoma cancer [57–59]. Moreover, increased expression of autophagy markers was found to be correlated with poor survival and radiotherapy/chemotherapy response in astrocytoma patients [60]. Additionally, PKCε has been implicated in the regulation of both cell survival and apoptosis in various cellular systems. In our study, the correlation between PKCε and autophagy was confirmed with downregulation of autophagy related genes after silencing PKCε. Thereafter, we found that knockdown of PKCε induced a decrease in the expression of Beclin1, Atg5, PI3K, whereas the expression of other autophagy-related proteins mTOR and Bcl2 was increased. Obtained data thus far suggest a connection between PKCε expression and autophagic pathway.

It is important to include autophagy modulators due to the assessment of changes in autophagy pathways. To delineate the molecular mechanisms involved in the autophagy interruption induced by silencing of PKCε, we examined the influence of positive autophagy regulator rapamycin. Treatment of control siRNA glioma cells with that activator induced autophagosome formation and an increase of LC3-II level and caused a decrease in the expression of p62. In contrast, in rapamycin-treated PKCε siRNA cells, we did not observe an increase in the presence of autophagic vacuoles and changes in LC3-II and p62 protein levels.

Moreover, in our studies, we discovered that the silencing of *PRKCE* gene and specifically decline of the expression of PKCɛ caused a significant diminution in the Akt phosphorylation of Ser in position 473 and expression of Akt protein level in both glioblastoma cells. The serine/threonine kinase Akt is widely acknowledged as a proto-oncogene. It is an essential component in PI3K/Akt/mTOR pathway. The PI3K/Akt/mTOR pathway is a crucial regulator of cell growth, including proliferation, growth, differentiation, and survival. Therefore, abnormal activation of PI3K/Akt/mTOR signaling pathway is commonly involved in the development and progression of various tumors [61]. The activation of PI3K phosphorylates and activates Akt, which have numerous downstream effects including activating mTOR [62]. Studies by Yang F and Gao JY showed that inhibition of PI3K/Akt/mTOR pathway by an inhibitor (BEZ235) or PI3KCA knockdown (shRNA transduction) significantly suppressed cell proliferation, migration, invasion and induced apoptosis human colon cancer cells [63].

The FAK protein is overexpressed in many cancers, including neuroblastoma, glioblastoma, breast cancer, colorectal cancer, pancreatic cancer, lung cancer, ovarian cancer [64, 65]. FAK autophosphorylation at Tyr397 (Y397 FAK) creates a binding site for c-Src that phosphorylates FAK at Tyr576 (Y576 FAK) and Tyr577 (Y577 FAK), promoting maximal FAK catalytic activity. In fact, FAK phosphorylation at Y576 and Y577 is required for maximal Y397 phosphorylation [66]. Our studies showed that PKCε downregulation caused reduction of adhesion of U-138 MG and U-118 MG cells by diminished activation and level of FAK. Many studies show that cancer cells with reduced potential for adhesion consequently lose functional links with the extracellular matrix. Therefore, they cannot move and adhere to other locations, which prevent the development of metastases [67, 68]. Studies by Nam JK and co-workers confirm that histone deacetylase inhibitor suppresses cell migration and invasion in human glioma cells by inhibiting FAK/STAT3 signaling [67].

These results together with enhanced downregulation of autophagy-related genes in response to rapamycin-treatment in PKCε siRNA cells suggest that the expression of PKCε is essential for the autophagic signal transduction pathways. Due to the fact that GB cell lines not fully mimic GB tumors this subject needs further investigation [69]. Several studies have shown that upregulation of autophagy can function as a prosurvival pathway in the presence of chemotherapy and enhance tumor resistance to anticancer therapies [70, 71].

## Conclusion

In summary, our results identify an important role of PKCε in the autophagy and adhesion that may, more importantly, someday providing a novel therapeutic strategy witch improve the survival of patients with gliomas.

## Abbreviations

3-MA: 3-methyladenine
ATG: AuTophaGy-related genes
ATO: Arsenic trioxide
Dox: Doxycycline
GB: Glioblastoma
mTOR: mammalian target of rapamycin
PI3K: Phosphatidylinositol 3-kinase
PKCε: Protein kinase C epsilon
Rap: Rapamycin
TRAIL: TNF-related apoptosis-inducing ligand
